# Prevalence and perceptions of cell phone use while walking in high school students

**Published:** 2019-08

**Authors:** Jun Ren, Shumei Wang

**Affiliations:** ^*a*^School of Public Health, Fudan University, Shanghai, China.

## Abstract

**Background:**

The World Health Organization pointed out that in 2015, road traffic injuries were the first cause of death for children and adolescents aged 10 to 19 and one of the main causes of death for boys aged 10 to 14, resulting in the death of more than 110,000 children and adolescents. In the world, 38% of the children injured or killed on the roads are pedestrians every year. According to the blue book on Internet use and reading practice among Chinese minors (2017-2018), the overall penetration rate of Internet use among surveyed minors reached 98.1% by the end of 2017. The research report on road traffic injuries of children in China shows that most middle school students use electronic devices frequently when walking, which results in their distraction, thus affecting their personal safety. A large number of foreign literatures have proved that distracted walking can significantly increase the incidence of all kinds of dangerous behaviors of pedestrians, and the number of emergency cases caused by distracted walking with mobile phones and other electronic devices is on the rise. The use of electronic devices during walking has become an important factor threatening the safety of pedestrians. The aim of the present research is to investigate the current situation of cell phone use by high school students while walking, the occurrence of dangerous behaviors and the promotion factors, analyze the characteristics of high school students distracted walking, and provide guidance for targeted intervention.

**Methods:**

A total of 1,777 students from six senior high schools in Shanghai were selected for online questionnaire survey in December 2018 by convenience sampling. The questionnaire included basic demographic information and information related to distract walking.

**Results:**

In different road environments and walking states, the cell phones were most frequently used when waiting for traffic lights and in the residential roads, and 21.3% of students often, frequently or always used mobile phones in these two situations. Listening to music or e-books was the main use of mobile phones when walking, and 27.3% of students often, frequently or always listened to music when walking. The information acquisition (navigation, etc.) was the most important promotion factor, with an average degree of influence of 4.38 (total score of 7). 54.1% of the students had some dangerous behaviors such as not paying attention to traffic lights and road conditions due to using mobile phones, and 41.7% of the students reported that the occurrence of sprained feet, tripping and collisions in the past was related to distract walking. In terms of the impact of different ways of using mobile phones on travel safety, the score of playing games (5.67) was about twice as high as that of listening to music (3.23) (the total score is 7).

**Conclusions:**

At present, high school students' distracted walking phenomenon is serious. It is very important to understand the factors influencing the use of mobile phones by students while walking, to carry out the set of school education, parents’ education, peer education and social education as one of the pedestrian safety education, and to strengthen students' awareness of the dangers of distracted walking.

**Keywords:**

Distraction, Pedestrian, Accidents, Traffic, High school, Students

